# *Wisteria floribunda* Agglutinin and Its Reactive-Glycan-Carrying Prostate-Specific Antigen as a Novel Diagnostic and Prognostic Marker of Prostate Cancer

**DOI:** 10.3390/ijms18020261

**Published:** 2017-01-26

**Authors:** Kazuhisa Hagiwara, Yuki Tobisawa, Takatoshi Kaya, Tomonori Kaneko, Shingo Hatakeyama, Kazuyuki Mori, Yasuhiro Hashimoto, Takuya Koie, Yoshihiko Suda, Chikara Ohyama, Tohru Yoneyama

**Affiliations:** 1 Department of Urology, Hirosaki University Graduate School of Medicine, Hirosaki 036-8562, Japan; hagiwara.kazuhisa@gmail.com (K.H.); tobisawa@hirosaki-u.ac.jp (Y.T.); shingoh@hirosaki-u.ac.jp (S.H.); moribio@hirosaki-u.ac.jp (K.M.); goodwin@hirosaki-u.ac.jp (T.K.); coyama@hirosaki-u.ac.jp (C.O.); 2 Corporate R&D Headquarters, Konica Minolta, Inc., Hino-shi, Tokyo 191-8511, Japan; takatoshi.kaya@konicaminolta.com (T.K.); tomonori.kaneko1@konicaminolta.com (T.K.); yoshihiko.suda@konicaminolta.com (Y.S.); 3 Department of Advanced Transplant and Regenerative Medicine, Hirosaki University Graduate School of Medicine, Hirosaki 036-8562, Japan; bikkuri@opal.plala.or.jp

**Keywords:** prostate-specific antigen, *N*-glycan, LacdiNAc, *Wisteria floribunda* agglutinin (WFA) lectin, biomarker

## Abstract

*Wisteria floribunda* agglutinin (WFA) preferably binds to LacdiNAc glycans, and its reactivity is associated with tumor progression. The aim of this study to examine whether the serum LacdiNAc carrying prostate-specific antigen–glycosylation isomer (PSA-Gi) and WFA-reactivity of tumor tissue can be applied as a diagnostic and prognostic marker of prostate cancer (PCa). Between 2007 and 2016, serum PSA-Gi levels before prostate biopsy (Pbx) were measured in 184 biopsy-proven benign prostatic hyperplasia patients and 244 PCa patients using an automated lectin-antibody immunoassay. WFA-reactivity on tumor was analyzed in 260 radical prostatectomy (RP) patients. Diagnostic and prognostic performance of serum PSA-Gi was evaluated using area under the receiver-operator characteristic curve (AUC). Prognostic performance of WFA-reactivity on tumor was evaluated via Cox proportional hazards regression analysis and nomogram. The AUC of serum PSA-Gi detecting PCa and predicting Pbx Grade Group (GG) 3 and GG ≥ 3 after RP was much higher than those of conventional PSA. Multivariate analysis showed that WFA-reactivity on prostate tumor was an independent risk factor of PSA recurrence. The nomogram was a strong model for predicting PSA-free survival provability with a *c*-index ≥0.7. Serum PSA-Gi levels and WFA-reactivity on prostate tumor may be a novel diagnostic and pre- and post-operative prognostic biomarkers of PCa, respectively.

## 1. Introduction

Prostate cancer (PCa) is a common cancer in men worldwide [1,2]. The most important issues regarding PCa is overdiagnosis and overtreatment [3,4]. Although the majority of patients diagnosed as clinically localized PCa, 30%–40% of patients who receive aggressive treatment such as radical prostatectomy (RP) experience biochemical recurrence [5,6]. Although, active surveillance (AS) is also proposed for low-risk PCa patients who meet the Prostate Cancer Research International Active Surveillance (PRIAS) criteria, 10%–30% of AS patients experience extraprostatic extension, and 42%–80% of AS patients experience an upgrade of the Gleason score after RP (ope GS) [7,8,9,10]. Pre-operative prostate-specific antigen (PSA) levels and biopsy GS are also powerful indicators of biological outcomes after RP [11]. Nevertheless, these indicators are not sufficient to prevent the overtreatment of PCa, and there is a need for more accurate diagnostic and prognostic indicators to select an appropriate treatment option for localized PCa.

**Figure 1:**
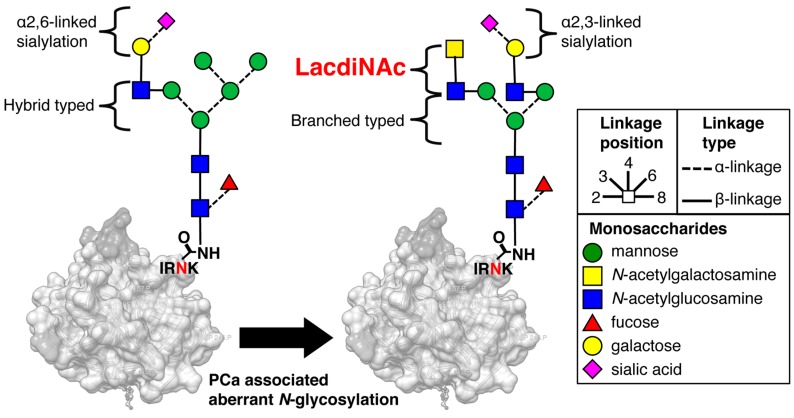
Prostate cancer (PCa)-associated aberrant *N*-glycosylation of prostate-specific antigen (PSA). PSA derived from PCa serum and culture supernatant of LNCaP carries *Wisteria floribunda* agglutinin (WFA)-reactive LacdiNAc glycans; this is not the case for PSA derived from benign prostatic hyperplasia (BPH) serum. PCa-associated aberrant LacdiNAc carrying PSA glycosylation isomer designated as PSA–glycosylation isomer (PSA-Gi) [21]. Carbon linkage positions are denoted by the bond position drawn on each monosachharide. IRNK indicate *N*-glycosylation site of PSA.

**Figure 2:**
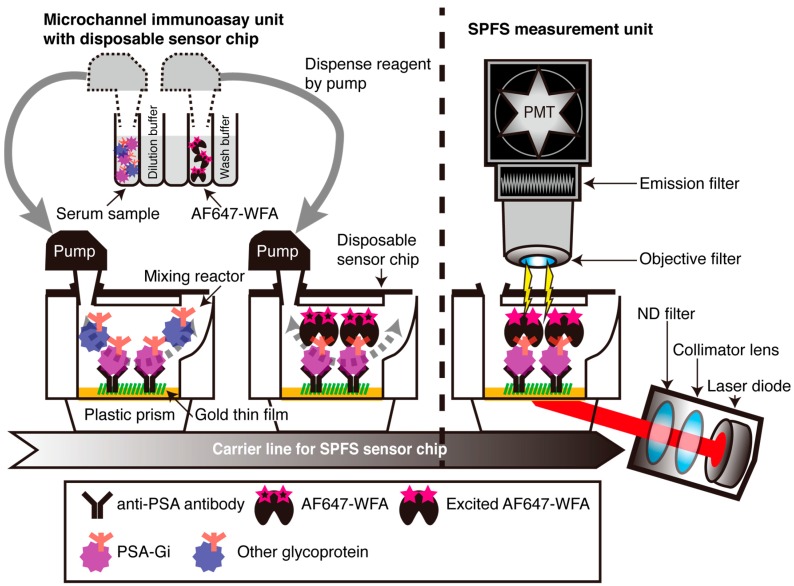
The schematic representation of serum PSA-Gi detection using a two-step surface plasmon field-enhanced fluorescence spectrometry (SPFS)-based WFA lectin-anti-PSA antibody immunoassay. Gray line arrows indicated that reagent dispense from reagent container to mixing reactor using pump. Gray dotted line arrows indicated mixing the content of mixing reactor by pump.

*N*- and *O*-glycosylation plays important roles in disease progression. The nonreducing terminal GalNAcβ1-4GlcNAc-(LacdiNAc) structure is found in *N*- and *O*-glycans of many mammalian glycoproteins though in very small amounts [12]. *Wisteria floribunda* agglutinin (WFA) is a good probe for LacdiNAc glycan [12]. Several researchers reported about LacdiNAc expression in cancer using WFA. They stated that LacdiNAc in *N*-glycans significantly decreases during progression of human breast cancer [13,14]. In contrast, the enhanced expression of LacdiNAc has been shown to be associated with the progression of human prostate, ovarian, colon, and liver cancers [12,15,16,17]. Therefore, the quantification of LacdiNAc glycan carrying glycoproteins or tissue-specific expression of LacdiNAc glycan detected by the WFA has shown promise as cancer glycobiomarkers [17,18,19]. In particular, regarding PCa, there are only three papers about LacdiNAc distribution in prostate biopsy (Pbx) and RP specimens using WFA [15,16,20], and they did not report the relation between WFA-reactivity in tissues and PCa prognosis. Although there are only a few reports including our group’s about PCa-associated aberrant LacdiNAc carrying PSA-glycosylation isomer (PSA-Gi) (Figure 1) [21,22], we demonstrate a pilot study of serum PSA-Gi as a diagnostic biomarker by using an automated two-step WFA–anti-PSA antibody sandwich immunoassay using high-sensitivity surface plasmon field-enhanced fluorescence spectrometry (SPFS) (Figure 2) [22]. Therefore, in this study, we retrospectively evaluated diagnostic and pre-operative prognostic performance of serum PSA-Gi and examined the association between WFA-reactivity on PCa tissues and PSA recurrence after RP.

## 2. Results

### 2.1. Diagnostic Performance of Serum PSA-Gi before Pbx Much Superior to Total PSA

**Figure 3:**
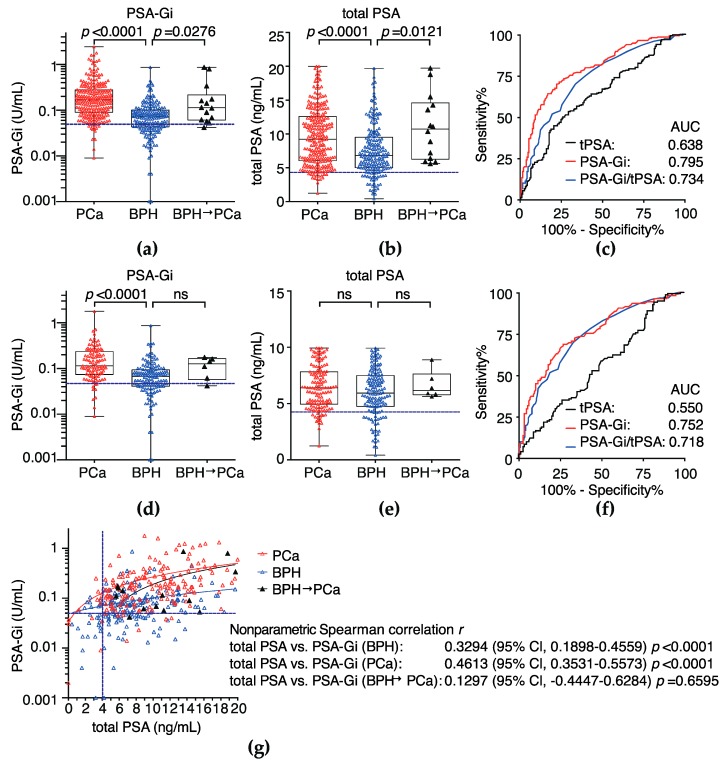
Serum levels of the PSA-Gi at Pbx in the patients who diagnosed as BPH or PCa by an SPFS-based lectin-antibody immunoassay. (**a**) PSA-Gi and (**b**) total PSA levels in patients with a diagnosis of BPH or PCa at a total PSA ≤ 20 ng/mL; (**c**) receiver-operator characteristic (ROC) curve analysis of total PSA, PSA-Gi, and PSA-Gi/total PSA in patients who had a diagnosis of BPH or PCa at a total PSA ≤ 20 ng/mL. The areas under the ROC curve (AUCs) for the prediction of PCa of PSA-Gi, total PSA, and PSA-Gi/total PSA were 0.795, 0.638, and 0.734, respectively; (**d**) PSA-Gi and (**e**) total PSA levels in patients with BPH or PCa at total PSA ≤ 10 ng/mL; (**f**) ROC curve analysis of total PSA, PSA-Gi, and PSA-Gi/total PSA in patients with BPH or PCa at a total PSA ≤ 10 ng/mL. The AUCs for the prediction of PCa by means of PSA-Gi, total PSA, and PSA-Gi/total PSA were 0.752, 0.550, and 0.718, respectively; (**g**) correlation between PSA-Gi and total PSA. Correlation coefficient was analyzed by non-parametric Spearman’s *r*-test. (**a**–**g**) The cutoff level at 90% sensitivity of PSA-Gi and/or total PSA is presented as a blue dotted line.

Serum PSA-Gi levels before Pbx was measured in patients with benign prostatic hyperplasia (BPH) (*n* = 184) or PCa (*n* = 244) to evaluate diagnostic performance. Patients’ characteristics in the BPH and PCa groups are shown in Table 1. Serum PSA-Gi levels in the both total PSA range ≤20 ng/mL (Figure 3a,b) and ≤10 ng/mL (Figure 3d,e) were significantly higher in patients with PCa (median: 0.1680 U/mL and median: 0.1140 U/mL, respectively) than in patients with BPH (median: 0.0715 U/mL and median: 0.0670 U/mL, respectively), *p* < 0.0001. The area under the receiver-operator characteristic curve (AUC) of PSA-Gi predicting PCa in any concentration range of total PSA (0.795, 95% CI; 0.753–0.837 and 0.752, 95% CI; 0.690–0.813, respectively) was much higher than those of PSA-Gi/total PSA (0.734, 95% CI; 0.686–0.782 and 0.718, 95% CI; 0.659–0.779, respectively) and total PSA (0.638, 95% CI; 0.586–0.691 and 0.550, 95% CI; 0.483–0.618, respectively) (Table 2, Figure 3c,f). At the cutoff PSA-Gi levels (0.0495 U/mL) for the prediction of PCa, the specificity at 90% sensitivity was 36.8%—much higher than the specificity of total PSA (18.8%). Furthermore, we found that higher PSA-Gi levels (≥0.1140 U/mL) in patients with BPH at first Pbx moderately predicted a diagnosis of PCa within 1–4 years after the first Pbx (Figure 3a,d). The nonparametric spearman correlation coefficient between the PSA-Gi level in BPH and total PSA in BPH was 0.3294 (95% CI, 0.1989–0.4559, *p* < 0.0001) and that between the PSA-Gi level in PCa and total PSA in PCa was 0.4613 (95% CI, 0.3531–0.5573, *p* < 0.0001) (Figure 3g). This means the PSA-Gi level was positively correlated with total PSA in BPH and PCa patients.

### 2.2. Serum PSA-Gi before Pbx Can Discriminate between Pbx Grade Group 2 and 3

**Figure 4:**
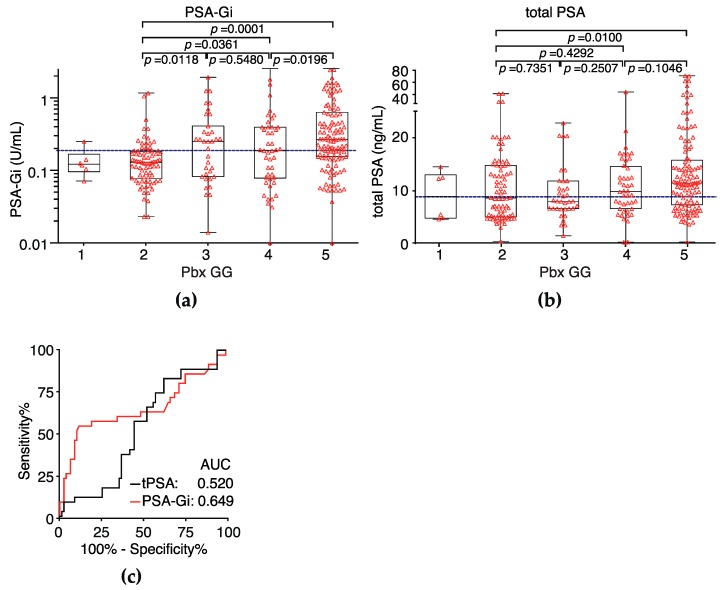
The serum PSA-Gi levels at Pbx in PCa patients who underwent radical prostatectomy (RP). (**a**) PSA-Gi levels before Pbx among PCa patients classified by the Pbx grade group (Pbx GG); (**b**) total PSA level before Pbx of PCa patients classified by the Pbx GG. Cutoff levels at 57.1% sensitivity of PSA-Gi and/or total PSA is presented as a blue dotted line; (**c**) ROC curve analysis of total PSA and PSA-Gi in PCa patients with Pbx GG 2 and Pbx GG 3. The AUCs for the prediction of patients with Pbx GG 3 of PSA-Gi and total PSA were 0.649 and 0.520, respectively.

Serum PSA-Gi levels before Pbx was measured in 244 PCa patients to evaluate the pre-operative predictor for a prostate biopsy. PSA-Gi levels were significantly correlated with Pbx grade group (GG) [23] (Figure 4a,b). Although total PSA could not discriminate between Pbx GG 2 and 3, serum PSA-Gi levels were significantly higher at ope GG 3 (median: 0.2500 U/mL, *p* = 0.0118) than at ope GG 2 (median: 0.1280 U/mL, Figure 4a,b). The AUC of PSA-Gi predicting Pbx GG 3 tumors was 0.649 (95% CI, 0.5221–0.7735) in contrast to the total PSA AUC of 0.520 (95% CI, 0.4091–0.6312; *p* = 0.162; Figure 4c). At the cutoff PSA-Gi level (0.1930 U/mL) for the prediction of GG 3 tumors at Pbx, sensitivity was 57.1%, and specificity was 80.8%—muchhigher than the specificity of the total PSA test (47.4%).

### 2.3. Serum PSA-Gi before Pbx Can Discriminate between Ope Grade Group ≤2 and ≥3

**Figure 5:**
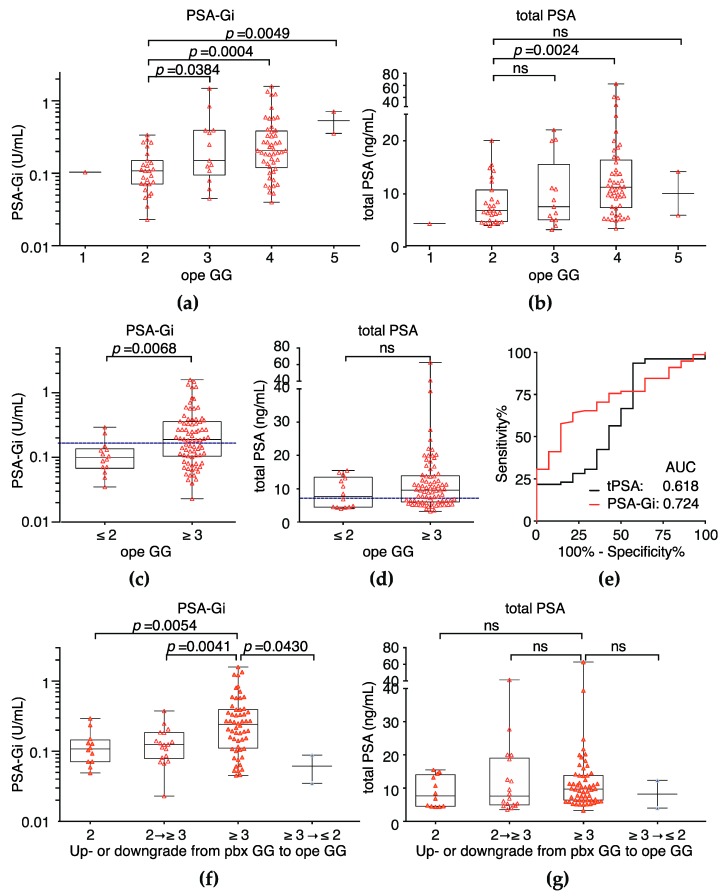
The serum PSA-Gi levels at Pbx in PCa patients who underwent RP. (**a**) PSA-Gi levels before Pbx among PCa patients classified by the grade group after RP (ope GG); (**b**) total PSA level before Pbx of PCa patients classified by the ope GG; (**c**,**d**) PSA-Gi and total PSA levels before Pbx between patients with ope GG ≤ 2 and ope GG ≥ 3. Cutoff levels at 60% sensitivity of PSA-Gi and/or total PSA is presented as a blue dotted line; (**e**) ROC curve analysis of total PSA and PSA-Gi in PCa patients with ope GG ≤ 2 and ope GG ≥ 3. The AUCs for the prediction of patients with ope GG ≥ 3 of PSA-Gi and total PSA were 0.724 and 0.618, respectively; (**f**,**g**) PSA-Gi and total PSA levels in patients with a GG upgrade from 2 at Pbx to ope GG ≥ 3 and a GG downgrade from ≥3 at Pbx to ope GG ≤ 2.

Serum PSA-Gi levels before Pbx was measured in 92 PCa patients who underwent RP to evaluate the pre-operative prognostic performance. PSA-Gi levels were moderately correlated with grade group after RP (ope GG) [23] (Figure 5a,b). Although total PSA could not discriminate tumors with ope GG ≥ 3, serum PSA-Gi levels was significantly higher at ope GG ≥ 3 (median: 0.1885 U/mL, *p* = 0.0068) than at ope GG ≤ 2 (median: 0.0985 U/mL, Figure 5c,d). The AUC of PSA-Gi predicting ope GG ≥ 3 tumors was 0.724 (95% CI, 0.603–0.845) in contrast to the total PSA AUC of 0.618 (95% CI, 0.442–0.794; *p* = 0.202; Figure 5e). Furthermore, the PSA-Gi levels tended to be higher in patients with a GG upgrade from 2 at Pbx to ope GG ≥ 3 and were associated with a GG downgrade from ≥3 at Pbx to ope GG ≤ 2 (Figure 5f,g). At the cutoff PSA-Gi level (0.1445 U/mL) for the prediction of GG ≥ 3 tumors, sensitivity was 60.3%, and specificity was 78.6%—much higher than the specificity of the total PSA test (50.0%).

### 2.4. Tumors Strongly and Moderately Positive for WFA Is an Independent Risk Factor of PSA Recurrence

**Figure 6:**
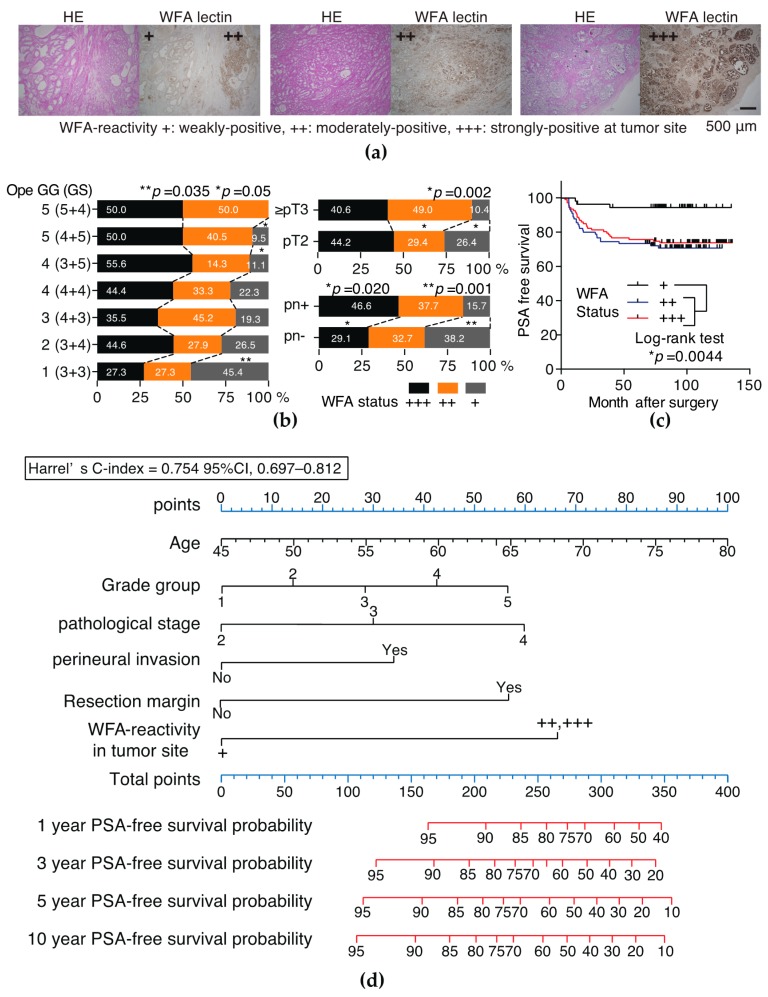
Immunohistochemical analysis of RP specimens using WFA lectin and post-operative nomogram predicting PSA-free survival probability. (**a**) Representative hematoxylin-eosin (HE) staining and WFA reactive-glycan expression of tumors of RP specimens. WFA-reactivity was classified into three groups: weakly positive, moderately positive, and strongly positive at a tumor site, respectively. Scale bar indicated 500 μm; (**b**) association between with WFA-reactive glycan expression and ope GG (ope GS), pathological stage, and perineural invasion-status; (**c**) PSA-free survival was evaluated using Kaplan–Meier curves and differences between three groups were assessed using the log-rank test. Patients with tumors strongly or moderately positive for WFA had a much shorter period of PSA recurrence after RP than did patients with tumors weakly positive for WFA; (**d**) Cox hazard regression analysis-based post-operative nomogram predicting PSA-free survival probability after RP. The c-index (0.754, 95% CI, 0.697–0.812), which is similar to the area under a receiver operating characteristic curve, was used to estimate the discrimination ability of the nomogram [25].

**Figure A1:**
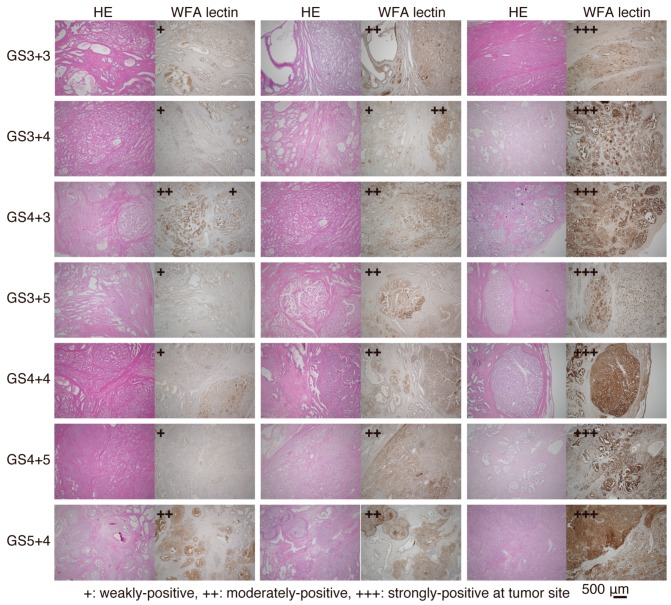
Representative hematoxylin-eosin (HE) staining and WFA reactive-glycan expression of tumors of each Gleason grade among RP specimens. WFA staining intensity was classified into three groups: weakly positive, moderately positive, and strongly positive at a tumor site, respectively. Scale bar indicated 500 μm.

Immunohistochemical staining of RP specimens by WFA was performed to examine the association between WFA-reactivity of tumor site and clinicopathological status. Patients’ characteristics in the 260 RP patients are shown in Table 3. WFA-reactive glycan was expressed in both benign prostate glands and tumors. On the basis of the reciprocal intensity of a tumor site [24], the WFA-reactivity was classified into three groups: weakly positive (median 78.5, range 74–85), moderately positive (median 98.5, range 86–104), and strongly positive (median 132, range 105–170; Figure 6a and Figure A1). When collated with these criteria, tumors strongly and moderately positive for WFA were significantly associated with a higher ope GS, pathological stage (≥pT3), and perineural invasion (pn)-positive status (Figure 6b and Table 3). As shown in Figure 5c, patients with tumors strongly and moderately positive for WFA had a much shorter period of PSA recurrence after RP than patients with tumors weakly positive for WFA (log-rank test, *p* = 0.0044). Multivariate Cox regression analysis revealed that WFA-reactivity was an independent risk factor of PSA recurrence (Table 4) and developed post-operative nomogram including WFA-reactivity, age, grade group, pT, RM, and pn status for prediction of PSA-free survival provability (Figure 6d). The c-index of nomogram was 0.754 (95% CI, 0.697–0.812) [25].

## 3. Discussion

One of the most important problems with PCa is overdiagnosis [3]. PSA-based screening has become controversial due to false positive results of total PSA in the PSA gray zone [4]. Overtreatment is also a major problem among certain segments of PCa patients [3] such as localized PCa and active surveillance patients [7,8,9,10]. Current biomarkers are not sufficient to prevent the overtreatment of PCa. Several serum-based testing (Phi, %p2PSA, and 4KScore), urine-based testing (PCA3) and MRI imaging has shown promising results in terms of diagnosis, localization, risk stratification, and staging of clinically significant PCa [26,27]. However, these promising biomarkers and imaging data are not yet cost-effective enough for routine clinical practice [28]. Therefore, there is a need for more accurate and cost-effective diagnostic and prognostic biomarkers. PCa-associated aberrant glycosylation of PSA is one of the candidate biomarkers. Fukushima et al. demonstrated that PSA derived from PCa serum and culture supernatant of LNCaP carries WFA-reactive LacdiNAc glycans; this is not the case for PSA derived from BPH serum [21] (Figure 1).

In the present study, we evaluated the diagnostic and pre-operative prognostic performance of WFA-reactive glycan-carrying PSA-Gi by using an SPFS-based automated immunoassay system [22]. We demonstrated that the AUC of PSA-Gi predicting PCa was much higher than that of the total PSA and PSA-Gi/total PSA (Figure 3c,f). We also demonstrated that a higher PSA-Gi level in BPH patients was moderately associated with a diagnosis of PCa within 1–4 years after first biopsy (Figure 3a,d). These results suggested that the diagnostic performance of a PSA-Gi single marker was much superior to conventional total PSA. 

Furthermore, we showed that PSA-Gi before Pbx significantly higher in patients with Pbx GG 3 than that of patients with Pbx GG 2 and specificity for prediction of Pbx GG 3 was much higher than PSA (Figure 4a–c). This suggests that PSA-Gi can discriminate between GG 2 and GG 3 tumors and may be used as a predictor for a prostate biopsy to discriminate between non-aggressive and aggressive tumors in the active surveillance program. We also showed that the AUC of PSA-Gi predicting ope GG ≥ 3 tumors was higher than that of the total PSA and specificity for prediction of ope GG ≥ 3 was much higher than PSA (Figure 5e). The PSA-Gi levels before Pbx tends to be higher in patients with GG upgraded from 2 at Pbx to ope GG ≥ 3. A similar result was reported that pre-operative fucosylated haptoglobin (Fuc-Hpt) levels is significantly higher in patients with GS ≥ 7 than those with GS ≥ 6 [29]. Nevertheless, the serum Fuc-Hpt levels is also higher in patients with pancreatic, ovarian, and hepatocellular cancers [30,31]. In addition, Li et al. reported that the serum fucosylated PSA (Fuc-PSA) levels is significantly higher in patients with GS ≥ 7 than those with GS ≥ 6 [32]. It is well-known that PSA is a prostate-specific protein, and aberrant glycosylation of PSA including Fuc-PSA and PSA-Gi was thus found to be a more specific glycobiomarker of PCa than Fuc-Hpt. Although our sample size is small and retrospective, these results suggest that aberrant glycosylation of PSA is associated with PCa aggressiveness. Stark et al. demonstrated that GG 3 tumors are associated with a three-fold increase in lethal PCa compared with GG 2 tumors in RP specimens [33]. More recently, Epstein et al. also demonstrated that there are large differences in 5-year recurrence rates between both the GG 2 and GG 3 in a large multi-institutional surgical cohort and hazard ratios for GG 3 disease were generally threefold higher than for GG 2 [34]. Therefore, discrimination between GG 2 and GG 3 is an important task for the reduction of overtreatment of PCa. Thus, our PSA-Gi may be a promising pre-operative prognostic biomarker predicting Pbx GG 3 tumors and ope GG ≥ 3 tumors, particularly in very low-risk PCa patients who have met PRIAS criteria and PCa patients at an intermediate risk.

Moreover, we examined WFA-reactivity of prostate tumors showed that tumors strongly and moderately positive for WFA are significantly associated with higher ope GG, pT, and pn-positive status (Figure 6b) and worse PSA-free survival as compared to patients with weakly positive tumors for WFA (Figure 6c). Cox regression analysis here provided WFA-reactivity in tumors was an independent risk factor of PSA recurrence (Table 4). Thus, nomogram developed in this study including WFA-reactivity in the tumor site combined with clinocopathological parameters seemed to be a strong model for predicting PSA-free survival provability with a c-index (0.754) (Figure 6d). Further internal and external validation study was required for the evaluation of predictive performance in this nomogram.

Our results reveal that serum PSA-Gi levels before Pbx is useful for the discrimination of PCa as well as Pbx GG 3 and ope GG ≥ 3 patients and the WFA-reactivity of tumors is also useful for the prediction of PSA recurrence. Thus, both PSA-Gi and WFA-reactivity of tumors may reduce overdiagnosis and overtreatment of PCa.

## 4. Materials and Methods

This study was performed in accordance with the ethical standards of the Declaration of Helsinki and was approved by the Ethics Committee of Hirosaki University Graduate School of Medicine (“The Study about Carbohydrate Structure Change in Urological Disease”; approval number: 2014-195; approval date: 22 December 2014). Informed consent was obtained from all patients.

### 4.1. Serum Samples from Patients with BPH and PCa

A total of 442 patients with benign prostatic hyperplasia (BPH) and PCa were treated at our hospital between June 2007 and August 2016. Serum samples from patients with BPH (*n* = 184), PCa (*n* = 244 of whom 92 patients underwent RP), or PCa who diagnosed as BPH at first Pbx (*n* = 14) were obtained before the first Pbx. The final diagnoses of BPH or PCa were confirmed using the histopathological findings of prostate biopsies. Staging and grading information of the tumors for RP patients was obtained from medical charts. The grade group of prostate biopsy and prostatectomy specimens were evaluated according to the International Society of Urological Pathology (ISUP) guidelines [23]. Patient demographics are shown in Table 1. All samples were stored at −80 °C until use.

### 4.2. Detection of Serum PSA-Gi and Total PSA

The serum PSA-Gi was detected by using an SPFS-based two-step WFA–anti-PSA antibody sandwich immunoassay with a disposable sensor chip as described previously [22]. The system was developed by Konica Minolta Inc. (Figure 1). Two-step sandwich SPFS immunoassays of PSA-Gi were carried out automatically by moving a cylindrical pump between the anti-total-PSA monoclonal antibody (No. 72, Mikuri Immunological Laboratories Co., Ltd., Osaka, Japan) immobilized on a thin gold film in a disposable sensor chip and a reagent container in a self-developed assay machine. The reagent container already contained a number of separate reagents, including wash buffer (TBS 0.05% Tween 20, 10× TBS (Nippon Gene Co., Ltd., Tokyo, Japan) and polysorbate 20 (MP Biomedicals, LLC., Santa Ana, CA, USA)), AF647-WFA (WFA (vector laboratories, Inc., Burlingame, CA, USA) labeled using an Alexa Fluor 647-labeling kit (A20186, Thermo Fisher Scientific Inc., Waltham, MA, USA)) and the sample for measurement. The 20 μL of serum was diluted by 100 μL of a PBS-based dilution buffe. Then the 100 μL diluted serum samples and AF647-WFA solution (10.0 μg/mL in 1% BSA in PBS) were allowed to react for 10 min, and unreacted lectins were removed with washing buffer (four washes) after the WFA lectin reaction. After four washes, the final washing buffer was kept for SPFS optical measurement in the microchannel of each disposable sensor chips. After the final washing step, AF647 in the microchannel of disposable sensor chips were sequentially excited by laser light, which was applied on the backside of a thin gold film through the plastic prism. The laser light was already p-polarized and collimated by the internal laser diode system. A laser diode (635 nm, 0.95 mW; Edmund Optics Japan, Ltd., Tokyo, Japan) was used as a light source with a Neutral Density filter (AND20C-10 (10%), Sigmakoki Co., Ltd., Saitama, Japan). The fluorescent signal of AF647 that passed through the emission filter (DIF-BP-1 (half width: 668 ± 5 nm), Optical Coatings Tokyo, Japan, Japan) was detected by a photomultiplier tube (H7421-40, Hamamatsu Photonics K.K., Shizuoka, Japan), which was located at the end of a light-converging optical system (numerical aperture, NA = 0.6; Edmund Optics Japan Ltd., Tokyo, Japan). All assays were conducted automatically at 25 °C; four immunoassays were carried out simultaneously. Standard PSA-Gi sample was obtained from culture supernatant of LNCaP cells (RCB2144, RIKEN Bio-resource Center through the National Bio-Resource Project of the MEXT, Tsukuba, Japan), as reported previously [22]. In brief, LNCaP cells were cultured in the RPMI 1640 medium (Thermo Fisher Scientific Inc., Waltham, MA, USA) supplemented with 10% fetal calf serum (FCS) at 5% CO_2_ at 37 °C. PSA secreted into the medium by the human PCa cell line, LNCaP cells, was used as a standard material of PSA-Gi in this study. The standard PSA-Gi concentration in the medium of the human LNCaP cell line was measured by WFA agarose column chromatography combined with a total-PSA enzyme-linked immunosorbent assay, as reported previously [22]. Fifty-five percent of total PSA in the medium of the LNCaP cell line possessed PSA-Gi (data not shown) [22]. Serum total PSA was measured by Architect i1000 system (Abbott Tokyo, Japan, Japan) and special reagents for total PSA (Abbott Japan) in a PSA range from 0.001 to 100 ng/mL.

### 4.3. Immunohistochemical Analysis of RP Specimens by WFA

A total of 260 paraffin-embedded RP specimens were obtained from PCa patients who underwent RP without neoadjuvant therapy between June 2007 and August 2016 in Hirosaki University Hospital. Patient demographics are shown in Table 3. Staging and grading information regarding the tumors and patient follow-up have been described previously [35]. In brief, PSA recurrence after RP was defined by two consecutive PSA values of >0.2 ng/mL with a 1-month interval and after a postoperative decrease below the detection limit (<0.001 ng/mL). Time zero was defined as the day of surgical treatment. Patients with constantly undetectable PSA levels (<0.001 ng/mL as the detection limit) after surgery were considered as patients without biochemical recurrence. Follow-up intervals were calculated from the date of the operation to the last recorded follow-up. Information on patients with PCa and tumor characteristics was obtained from medical charts. The grade group of prostate biopsy and prostatectomy specimens were evaluated according to the International Society of Urological Pathology (ISUP) guidelines [23]. Deparaffinized RP specimens were incubated with the biotinylated-WFA (Vector Laboratories, Burlingame, CA, USA) in PBS containing 5% of bovine serum albumin (1:500 dilution) at 4 °C, overnight. Biotinylated-WFA was detected by Vectastain Elite ABC kit (Vector Laboratories). WFA-reactivity was classified into three groups according to the reciprocal intensity scale as described previously [24]. Representative images of each Gleason grade tumor are shown in Figure A1.

### 4.4. Statistical Analysis

All calculations for clinical data were performed in the SPSS software, ver. 21.0 (SPSS, Inc., Chicago, IL, USA) and in GraphPad Prism 6.03 (GraphPad Software, San Diego, CA, USA). Intergroup differences were statistically analyzed by a Student’s *t*-test for normally distributed variables or by the Mann–Whitney *U*-test for non-normally distributed models. Data with *p* < 0.05 were considered significant. ROC curves developed using the library “rms” in R (available on: http://www.r-project.org/) [25] and the statistical difference of AUCs were calculated by the same program. The *χ*^2^ test was used to analyze the association of the WFA-reactivity status with clinicopathological parameters. PSA-free survival was evaluated using Kaplan–Meier curves, and differences between groups were assessed by the log-rank test. Multivariate test by Cox proportional hazards regression analysis was performed to detect significant and independent parameters with which PSA recurrence after RP can be predicted. Post-operative nomogram predicting PSA-free survival provability after RP was developed using the library “rms” in R (available on: http://www.r-project.org/), and the c-index was also calculated by same program [25].

## 5. Conclusions

At present, the majority of promising markers such as Phi, 4KScore, and tissue-based markers [26] are used in multiplex testing to improve diagnostic and prognostic accuracy. PSA-Gi is used as a single marker and yields results comparable to the diagnostic and prognostic performance of multiplex markers. PCA3 was also a promising urine marker for repeat biopsy decision-making [26]. However, there are a few cumbersome procedures for sample handling for avoiding RNA degradation. In this study, although we used frozen serum samples stored from 2007 to 2016, diagnostic and prognostic performance of PSA-Gi was substantially superior to total PSA. The serum sample handling of PSA-Gi was almost the same as the PSA test. Therefore, serum PSA-Gi is a promising pre-operative marker for detecting PCa and assessing the aggressiveness of PCa and has an advantage of cost-effectiveness and sample handling for routine clinical practice. Furthermore, the nomogram developed in this study is also a promising predictive tool for determining PSA-free survival probability. Larger clinical trials are warranted to confirm our findings.

## Figures and Tables

**Table 1:** Characteristics of BPH patients and PCa patients.

Characteristics	BPH ^a^	PCa ^b^		BPH-> PCa		*p* (^a^ vs. ^b^)
*n* = 442	184	244		14		
Age, median (range)	69 (30–87)	68 (44–85)		69 (52–80)		ns ^1^
PSA ^2^, ng/mL, median (range)	6.8 (0.4–19.7)	9.0 (1.2–62.6)		6.3 (5.9–19.7)		<0.001
PSA-Gi, U/mL, median (range)	0.0715 (0.001–0.86)	0.165 (0.002–2.43)		0.113 (0.04–0.87)		<0.001
PSA-Gi/total PSA, U/ng, median (range)	0.0100 (0.00–0.1150)	0.0200 (0.002–0.1980)		0.0135 (0.003–0.0640)		<0.001
Clinical T stage, *n* (%)		*n* = 244				
cT1		144	(59.3)			
cT2		46	(18.5)			
cT3		55	(22.2)			
Pbx GS ^3^, *n* (%) Pbx GG ^4^		*n* = 244				
3 + 3 ^1^		6	(2.4)			
3 + 4 ^2^		79	(32.4)			
4 + 3 ^3^		29	(11.9)			
4 + 4 ^4^		30	(12.3)			
3 + 5 ^4^		3	(1.2)			
4 + 5 ^5^		72	(29.5)			
5 + 4 ^5^		20	(8.2)			
5 + 5 ^5^		5	(2.0)			
Pathological T stage, *n* (%)		*n* = 92		*n* = 8		
pT1		4	(4.3)	0	(0)	
pT2		53	(57.6)	5	(62.5)	
pT3		38	(41.3)	3	(37.5)	
Ope GS ^5^, *n* (%) Ope GG ^6^		*n* = 92		*n* = 8		
3 + 3 ^1^		1	(1.1)			
3 + 4 ^2^		13	(14.1)	2	(25.0)	
4 + 3 ^3^		14	(15.2)			
3 + 5 ^4^		3	(3.2)	1	(12.5)	
4 + 4 ^4^		9	(9.8)	1	(12.5)	
5 + 3 ^4^		1	(1.1)			
4 + 5 ^5^		37	(40.2)	3	(37.5)	
5 + 4 ^5^		12	(13.0)	1	(12.5)	
5 + 5 ^5^		2	(2.2)			

^1^ not significantly difference; ^2^ total PSA; ^3^ prostate biopsy Gleason score; ^4^ prostate biopsy grade group; ^5^ Gleason score after radical prostatectomy; ^6^ grade group after radical prostatectomy. Pbx: prostate biopsy; ^a^ BPH; ^b^ PCa.

**Table 2:** Comparison of areas under the receiver-operator characteristic curve (AUCs) of PSA, PSA-Gi, and PSA-Gi/total PSA for the detection of PCa.

Test Name	PSA Range	AUC	95% CI	*p* (vs. ^a^)	*p* (vs. ^b^)	*p* (vs. ^c^)
Total PSA ^a^	-	0.638	0.586–0.691	-	<0.0001	0.0376
PSA-Gi ^b^	20 ng/mL	0.795	0.753–0.837	<0.0001	-	0.0003
PSA-Gi/total PSA^c^	-	0.734	0.586–0.691	0.0376	0.0003	-
Total PSA ^a^	-	0.550	0.483–0.618	-	<0.0001	<0.0001
PSA-Gi ^b^	10 ng/mL	0.752	0.690–0.813	<0.0001	-	0.567
PSA-Gi/total PSA ^c^	-	0.719	0.659–0.779	0.0009	0.0009	-

^a^ Total PSA test; ^b^ PSA-Gi test; ^c^ PSA-Gi/total PSA test.

**Table 3:** Characteristics of PCa patients who underwent RP categorized by WFA-reactivity.

Characteristics		WFA-Reactivity						*p*
		Weakly Positive ^a^		Moderately Positive ^b^		Strongly Positive ^c^		^a^ vs. ^b + c^
*n*, Total = 260		51		95		112		
Age, median (range)		68 (48–75)		68 (56–76)		68 (52–78)		0.555
PSA ^1^, ng/mL, median (range)		7.5 (2.3–18.4)		7.4 (0.6–27.6)		7.5 (0.5–35.9)		0.473
Pathological T stage, *n* (%)								0.008 ^2^
pT2, *n* = 163		41	(26.4)	48	(29.4)	72	(44.2)	0.002
pT3, *n* = 96		10	(10.4)	47	(49.0)	39	(40.6)	0.002
pT4, *n* = 1		0	(0)	0	(0)	1	(100)	0.612
Ope GS ^3^, *n* (%)	Ope GG ^4^							0.045 ^2^
3 + 3, *n* = 11	Ope GG ^1^	5	(45.4)	3	(27.3)	3	(27.3)	0.035
3 + 4, *n* = 112	Ope GG ^2^	28	(26.5)	34	(27.9)	50	(44.6)	0.108
4 + 3, *n* = 63	Ope GG ^3^	13	(19.3)	28	(45.2)	22	(35.5)	0.955
4 + 4, *n* = 9	Ope GG ^4^	2	(22.3)	3	(33.3)	4	(44.4)	0.889
3 + 5, *n* = 9	Ope GG ^4^	1	(11.1)	3	(33.3)	5	(55.6)	0.482
4 + 5, *n* = 42	Ope GG ^5^	4	(9.5)	17	(40.5)	21	(50.0)	0.056
5 + 4, *n* = 14	Ope GG ^5^	0	(0)	7	(50.0)	7	(50.0)	0.052
pn ^5^, *n* (%)								
pn−, *n* = 56		21	(37.5)	18	(32.1)	17	(30.4)	<0.001
pn+, *n* = 204		32	(15.7)	77	(37.7)	95	(46.6)	<0.001
RM ^6^, *n* (%)								
RM−, *n* = 188		43	(22.9)	65	(34.6)	80	(42.5)	0.108
RM+, *n* = 72		10	(13.9)	30	(41.7)	32	(44.4)	0.108
PSA failure, *n* (%)								
−, *n* = 194		49	(25.3)	66	(34.0)	79	(40.7)	<0.001
+, *n* = 66		4	(6.1)	29	(43.9)	33	(50.0)	<0.001

^1^ total PSA; ^2^ *χ*^2^ test; ^3^ Ope GS, Gleason score after radical prostatectomy; ^4^ Ope GG, grade group after radical prostatectomy; ^5^ pn, perineural invasion; ^6^ RM, resection margin; ^a^ weakly positive; ^b^ moderately positive; ^c^ strongly positive.

**Table 4:** Multivariate analysis to determine an independent predictor of PSA recurrence.

Variable	Hazard Ratio	Standard Error	*p*
Age	1.046	0.027	0.099
WFA-reactivity	2.831	0.529	0.049
pT ^1^	1.589	0.336	0.168
Grade group	1.246	0.099	0.027
RM ^2^	2.424	0.319	0.006
pn ^3^	1.715	0.447	0.227

^1^ pathological T stage; ^2^ resection margin; ^3^ perineural invasion.

## Abbreviations

SPFS	surface plasmon field-enhanced fluorescence spectrometry
WFA	*Wisteria floribunda* agglutinin
PSA	prostate-specific antigen
PCa	prostate cancer
BPH	benign prostatic hyperplasia
LacdiNAc	GalNAcβ1-4GlcNAc-
Gal	galactose
Man	mannose
Fuc	fucose
Sia	sialic acid
GalNAc	*N*-acetylgalactosamine
GlcNAc	*N*-acetylglucosamine
Pbx GS	prostate biopsy Gleason Score
Pbx GG	prostate biopsy grade group
cT	clinical T stage
pT	pathological T stage
Ope GS	gleason score after radical prostatectomy
Ope GG	grade group after radical prostatectomy
RP	radical prostatectomy
pn	perineural invasion
RM	resection margin
